# Percutaneous sclerosing injection to the thoracic duct under CT guidance for cervical chylous leakage post thyroidectomy: A case report

**DOI:** 10.1016/j.radcr.2021.06.054

**Published:** 2021-07-15

**Authors:** Nguyen Ngoc Cuong, Le Hoan, Le Tuan Linh, Pham Huy Tan, Thieu Thi Tra My, Nguyen Minh Duc

**Affiliations:** a Diagnostic imaging and interventional radiology center, Hanoi medical university hospital, Hanoi, Vietnam; b Respiratory medicine Department, Hanoi Medical University hospital, Ha Noi, Viet Nam; c Center of family medicine and healthcare, Hanoi Medical University hospital, Ha Noi, Viet Nam; d Department of Radiology, Ha Noi Medical University, Ha Noi, Viet Nam; e Department of Radiology, Pham Ngoc Thach University of Medicine, Ho Chi Minh City, Viet Nam

**Keywords:** Chylous leakage, Thoracic duct embolization, Thoracic duct sclerotic injection, Thyroidectomy

## Abstract

Chylous leakage after thyroidectomy is rare, and almost all patients with this complication can be treated conservatively. However, in patients with high-flow leakage, treatments can be complicated. In this study, we report a case that was successfully treated by disrupting the thoracic duct using two sessions of percutaneous interventions. The first intervention was a thoracic duct embolization, and the second intervention was a sclerosing injection to the thoracic duct under computed tomography guidance.

## Introduction

Chylous leakage (CL) after thyroidectomy is an extremely rare complication that affects approximately 0.9% of patients, regardless of whether cervical lymph node dissection is performed [1]. The leakage can occur immediately following the operation or a few days later when patients return to a normal diet [2]. Almost all cases of CL can be managed conservatively, through compression and the delivery of parenteral nutrition [2]. In patients for whom conservative treatment fails, prolonged CL can cause severe malnutrition, psychological depression, or even mortality [3].

The management of CL depends on the leakage volume and includes conservative and interventional treatment options [1]. A high-flow leak is classified as one in which the drainage volume exceeds 500 ml per day or for which the leakage volume does not decrease by >50% of the volume measured at the time of detection following 2 days of negative aspiration [1,3]. Interventional treatment should be indicated promptly upon the determination of a high-flow leak. Thoracic duct embolization (TDE) has been described as a potential treatment option that is less invasive than other treatments [4]. Catheterization into the thoracic duct to perform TDE was successful in 67% of patients in a large series of 109 patients [5]. When TDE attempts result in unsuccessful catheterization into the thoracic duct (TD), the needle interruption of the TD is recommended; however, the clinical success of needle interruption is not reliable. In this study, we highlight the role of a technique involving percutaneous thoracic puncture under CT guidance for the management of CL.

## Case report

A 42-year-old female patient was diagnosed with left lobe thyroid cancer. The patient underwent total thyroidectomy with cervical lymph node resection. Five days post-surgery, the cervical drain was producing 500 mL of milky fluid each day. The volume of the drain increased to 900 mL per day, even after the patient was treated with parenteral nutrition. The patient was referred to our hospital after two weeks of failed conservative treatment.

**Fig. 1:**
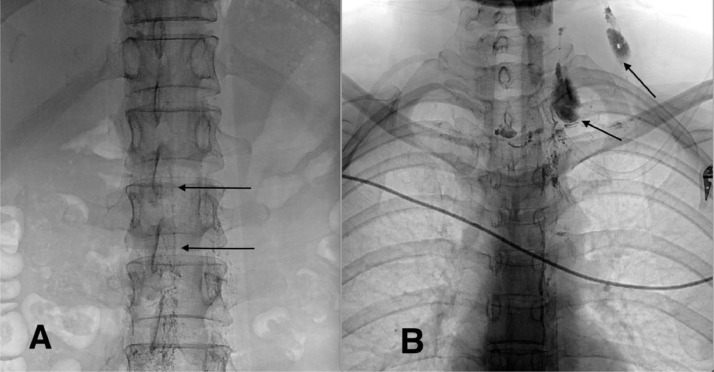
Lymphangiography showed the appearance of the cisterna chyli (A, arrows) and extravasation of the contrast agent to the distal part of the thoracic duct (B, arrows).

At our institution, following a multidisciplinary meeting, a decision was made to perform intranodal lymphagiography and TDE. A total of 10 ml lipiodol was slowly injected into the lymph nodes at the bilateral groin. This technique is well-known and has been described in detail by Nadolski and Itkin [6]. On lymphangiography, the contrast material appeared in the cisterna chyli and TD (Fig. 1A). The extravasation of the contrast agent at the distal part of the TD was also observed on lymphangiography (Fig. 1B).

**Fig. 2:**
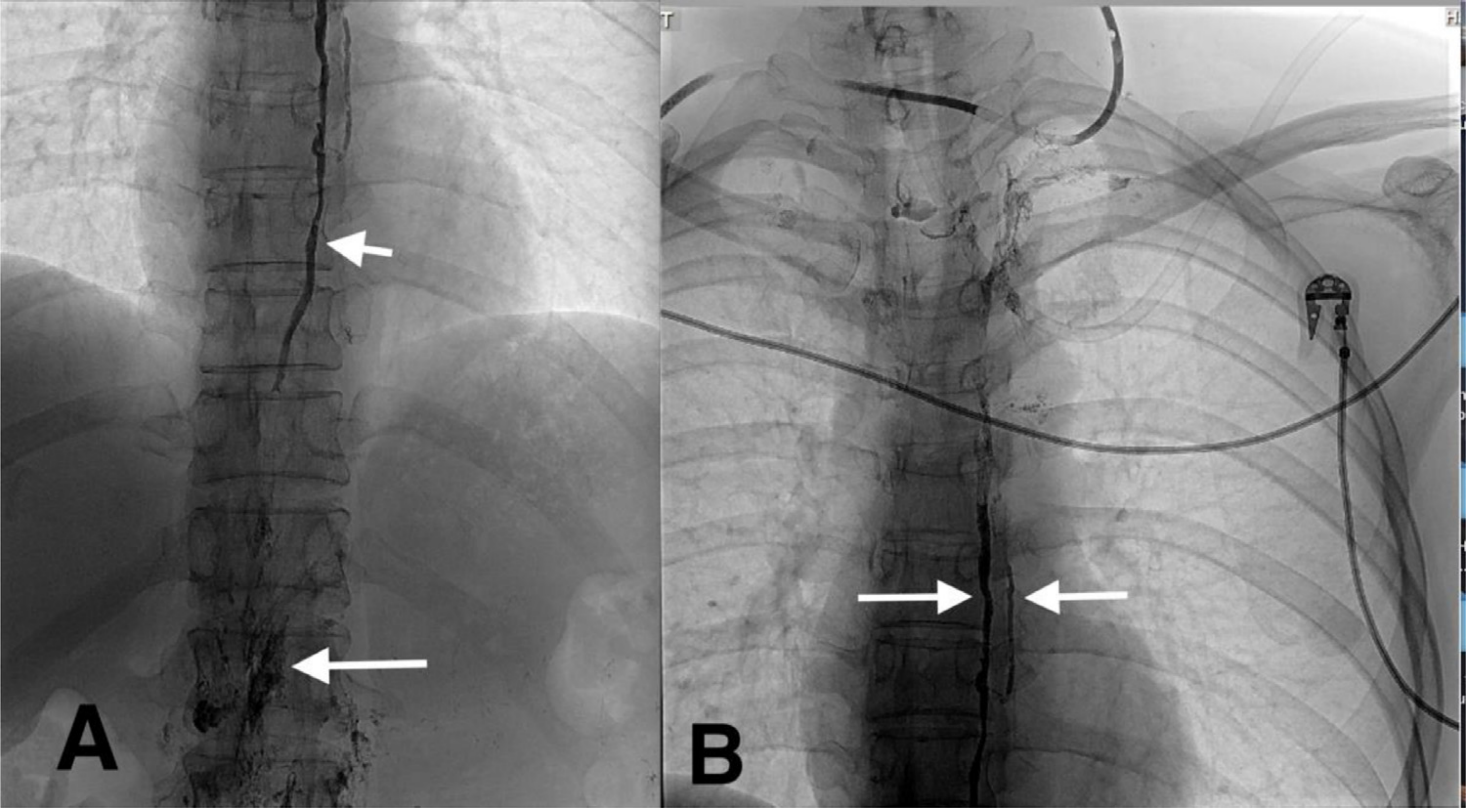
The glue cast was observed along the thoracic duct (TD). A) The glue was observed in the distal part of the TD (short arrow), and glue was observed in the retroperitoneal space (long arrow). B) The glue was observed in the middle segment of the TD and its branch (arrows).

The contrast agent was rapidly washed out at the abdominal level due to the inflow of non-contrast fluid from the intestinal and hepatic lymphatic vessels. After many failed attempts of puncturing the cisterna chyli with 21G needles, we successfully punctured the lower part of the TD using a 25-gauge needle (Chiba 25 G, Cook, USA). The tip of the needle was located in the TD, as confirmed by the injection of contrast material (Xenetix 350, Guerbet, Germany). After the contrast material was injected, the TD was visible, and the extravasation of a small amount of contrast agent was visible in the area surrounding the needle. The needle was flushed with 1 mL 5% glucose, and a 1.5 mL volume of a mixture consisting of N-butyl cyanoacrylate (NBCA) combined with lipiodol at a ratio of 1:3 was injected through the needle. Single short-exposure pictures after TDE showed the glue cast filling the TD, and glue was also observed in the retroperitoneal space (Fig. 2).

**Fig. 3:**
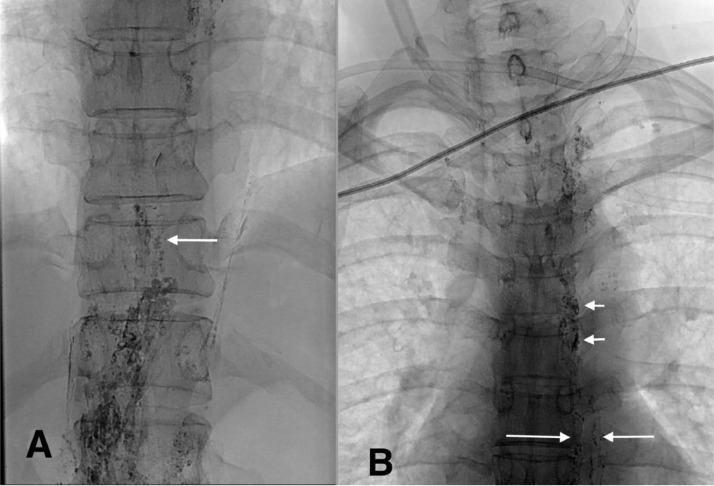
Radiography 8 days after thoracic duct (TD) embolization. A) Glue was completely washed out from the distal part of the TD (arrow). B) Some glue could be observed in the middle segment of the TD and its branch (long arrows), and minimal stagnation of the contrast agent was observed in the TD (short arrows).

After the TDE intervention, the drainage volume reduced significantly by up to 30–50 ml per day. However, the drainage volume increased again greater than 200 mL per day one week after TDE. Radiography performed 8 days after TDE showed only a small glue volume in the TD, indicating that the glue was being flushed into the thoracic laceration at the neck region (Fig. 3). We also noticed minimal stagnation of the contrast agent in the TD (Fig. 3B); therefore, we opted to puncture the TD under CT guidance.

**Fig. 4:**
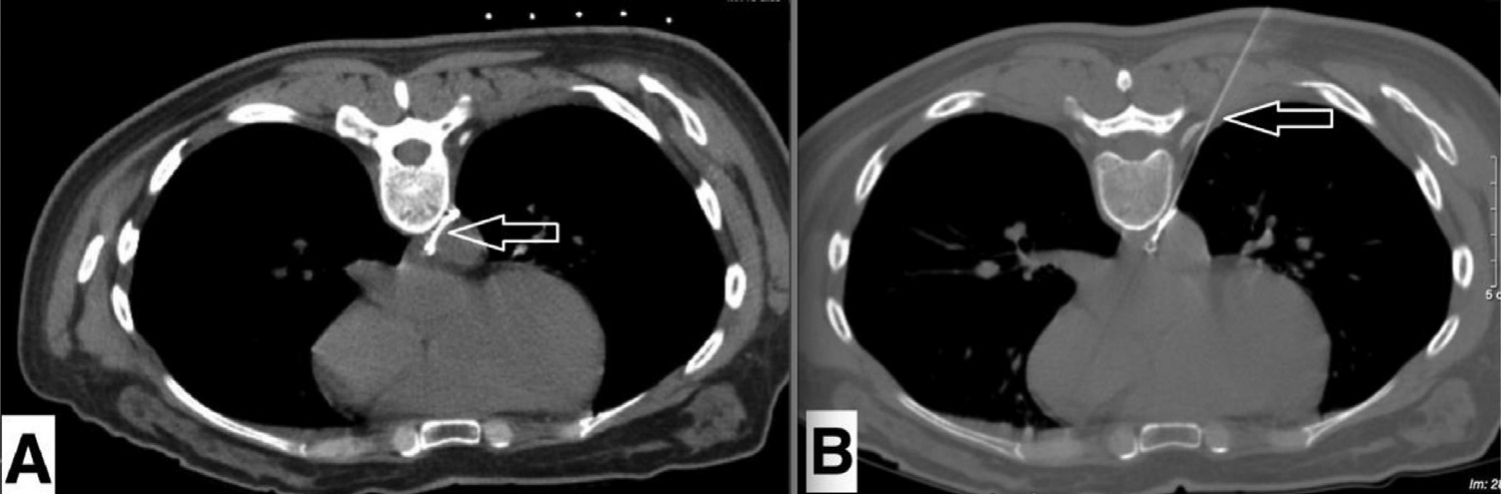
Chest CT scans 8 days after thoracic duct (TD) embolism. A) The TD, containing contrast agent, was observed (arrow). B) A needle was used to puncture the TD under CT guidance (arrow), and the sclerosing agent was injected into the TD.

The second intervention performed was sclerotherapy, 8 days following the first intervention. The patient was placed in a prone position under local anesthesia. A 25-gauge-needle was used to puncture the TD under CT guidance (Fig. 4). The tip of the needle was confirmed to be within the TD based on the CT image, and the aspiration of the needle extracted a creamy fluid. We injected 5 mL of a foam mixture consisting of polidocanol (aetoxisclerol 2%, Kreussler, France) and air, at a ratio of 1:4, into the TD. After the second intervention, the volume of the drain was reduced to under 10 mL per day. The drain was withdrawn after 3 days. The patient was discharged from the hospital, and no neck swelling was reported.

## Discussion

Thyroidectomy is a common operation that can lead to several complications, including bleeding, hypocalcemia, and recurrent laryngeal nerve injury, depending on the extent of the procedure [7]. TD injury is a rare and serious complication of this procedure. The overall incidence of CL in thyroidectomy ranges from 0.5% to 2.5% and is higher when associated with lymph node dissection, reaching 2%–8% [2,3]. Park *et al.* [1] reported an increased incidence of CL in patients with lateral neck dissection compared to those with central neck dissection.

The management of CL remains controversial, and no treatment guidelines exist for this complication [2]. Low-volume CL can be treated effectively with conservative management, including diet control, the application of drugs (somatostatin and octreotide), paracentesis, sclerotherapy, and pressure dressing [3]. However, patients with high-volume CL often require surgical intervention [2]. Lymphatic embolization is a minimally invasive intervention that can be used to avoid additional surgery [4]. TDE has become more common in the treatment of CL incidents, including those in the chylothorax and chylopericardium [8]. To date, only a few sporadic studies have described TDE performed in cases of CL after thyroid surgery [4].

TDE involves the transabdominal catheterization of the cisterna chyli and the embolization of the TD. Successful TDE requires the TD to be catheterized by a microcatheter, followed by the occlusion of the TD using a combination of coils and glue [5]. Chen and Itkin [8] reported a success rate for TDE of traumatic TD injuries of 91%. If TDE is unsuccessful, the percutaneous treatment of TD disruption is recommended [5]. TD disruption involves the disruption of the lymphatic vessels by repeated twisting or to-and-fro “twiddling” motions performed with a needle [9]. Another reported method for CL treatment was CT-guided sclerotherapy [10,11]. Sclerotherapy is typically used for the treatment of vascular and nonvascular diseases by disrupting the endothelium to generate fibrosis [12]. The therapeutic use of sclerosants, including ethanol, NCBA, and polidocanol, aims to disrupt the endothelium of the targeted structures. Kortes *et al.* [10] used ethanol injections into the surrounding space of a leakage point, and Garcial *et al.* [11] combined sclerotherapy with embolization to control CL. In these studies, the target needle position was placed as closely as possible to the leakage site, and the sclerosants were injected surrounding the leakage point, which was demonstrated to be an effective intervention for the treatment of CL.

Our patient presented with high-output CL and failed to respond to conservative management. To avoid the burden of reoperation, we opted to use the minimally invasive procedure of TDE. Although the first treatment appeared to be markedly effective immediately following the intervention, the amount of drainage increased significantly after 8 days. The cause of the recurrent CL appeared to be the failure to place a coil in the TD. The use of glue injected into the TD alone was not sufficient for a polymerization reaction capable of occluding the TD. Radiography confirmed that the glue was being washed out. In the second intervention, CT-guided sclerotherapy was utilized as another alternative therapy to avoid reoperation. TDE and CT-guided sclerotherapy are both less invasive and effective treatments compared with reoperation.

## Conclusion

In conclusion, high-output CL after thyroidectomy is a rare but severe complication. Percutaneous intervention represents a feasible solution to avoid reoperation. TDE should be considered as the first-line treatment for CL. In cases of TDE failure, CT-guided sclerotherapy could be an appropriate alternative option.

## Author declaration

We confirm that the manuscript has been read and approved by all named authors and that there are no other persons who satisfied the criteria for authorship but are not listed. We further confirm that the order of authors listed in the manuscript has been approved by all of us.
